# *Klebsiella pneumoniae* Biofilms and Their Role in Disease Pathogenesis

**DOI:** 10.3389/fcimb.2022.877995

**Published:** 2022-05-11

**Authors:** Maria Eduarda Souza Guerra, Giulia Destro, Brenda Vieira, Alice S. Lima, Lucio Fabio Caldas Ferraz, Anders P. Hakansson, Michelle Darrieux, Thiago Rojas Converso

**Affiliations:** ^1^ Laboratório de Biologia Molecular de Microrganismos, Universidade São Francisco, Bragança Paulista, Brazil; ^2^ Division of Experimental Infection Medicine, Department of Translational Medicine, Lund University, Malmo, Sweden

**Keywords:** biofilm, *Klebsiella pneumoniae*, quorum sensing, pathogenesis, virulence factors

## Abstract

The ability to form biofilms is a crucial virulence trait for several microorganisms, including *Klebsiella pneumoniae* – a Gram-negative encapsulated bacterium often associated with nosocomial infections. It is estimated that 65-80% of bacterial infections are biofilm related. Biofilms are complex bacterial communities composed of one or more species encased in an extracellular matrix made of proteins, carbohydrates and genetic material derived from the bacteria themselves as well as from the host. Bacteria in the biofilm are shielded from immune responses and antibiotics. The present review discusses the characteristics of *K. pneumoniae* biofilms, factors affecting biofilm development, and their contribution to infections. We also explore different model systems designed to study biofilm formation in this species. A great number of factors contribute to biofilm establishment and maintenance in *K. pneumoniae*, which highlights the importance of this mechanism for the bacterial fitness. Some of these molecules could be used in future vaccines against this bacterium. However, there is still a lack of *in vivo* models to evaluate the contribution of biofilm development to disease pathogenesis. With that in mind, the combination of different methodologies has great potential to provide a more detailed scenario that more accurately reflects the steps and progression of natural infection.

## Introduction

*Klebsiella pneumoniae* is a Gram-negative, encapsulated bacterium, responsible for a great variety of infections such as pneumonia, urinary tract infections, bacteremia, meningitis, and liver abscesses (Fang et al., 2000; Dao et al., 2014; Paczosa and Mecsas, 2016). The risk groups for *K. pneumoniae* infections includes newborns, the elderly, and immunocompromised individuals; however, the bacterium is also responsible for an increasing number of community acquired infections (Bengoechea and Sa Pessoa, 2019). The bacterium can be found in the environment (soil and superficial waters) and on abiotic surfaces such as medical instruments. It colonizes human mucosal surfaces (specially the oropharynx and the gastrointestinal tract) from where it may invade other tissues (Fang et al., 2000; Dao et al., 2014; Paczosa and Mecsas, 2016). In the last decade, there has been a great increase in the incidence of multidrug-resistant *K. pneumoniae* (Paczosa and Mecsas, 2016), highlighting the importance of a better understanding of *K. pneumoniae* pathogenesis.

*K. pneumoniae* strains are commonly classified as opportunistic, hypervirulent (hyKp) or multidrug-resistant (MDR) (Wang et al., 2020). While the classic *K. pneumoniae* (cKp) consist of opportunistic strains frequently associated with nosocomial infections, the hypervirulent strains are regarded as community acquired bacteria that can infect people of all ages, including healthy individuals (Chew et al., 2017; Russo and Marr, 2019). The rapid spread of multidrug-resistant *K. pneumoniae* strains is a major global health threat as these strains are responsible for a great number of hospital infections with high morbidity and mortality. In a recent study, Wyres and colleagues analyzed the genomic evolutionary, comparing MDR and invasive community-associated strains; they found that MDR clones present the greatest risk of infection, since they are more likely to acquire virulence genes than hypervirulent strains are to acquire resistance genes (Wyres et al., 2019).

An important virulence trait used by *K. pneumoniae* is its ability to form biofilms, bacterial communities containing one or more species, incorporated in an extracellular matrix composed by polysaccharide, proteins and DNA (Balestrino et al., 2005). Biofilm formation leads to increased resistance to exogenous stressors and antimicrobial factors (Brindhadevi et al., 2020; Wang et al., 2020). Considering the important role of biofilm formation for *K. pneumoniae* dissemination and virulence, the present review explores the bacterial factors involved in biofilm formation by this bacterium, the regulatory pathways controlling this mechanism, and various model systems used to study biofilm formation.

## Biofilm Formation and Function: An Overview

Biofilms are highly structured microbial communities that display increased resistance to antimicrobial factors and host defenses (e.g., the complement system, antimicrobial peptides, and phagocytosis). They have a highly complex and heterogeneous structure, composed of bacteria encased in an extracellular matrix made of proteins, carbohydrates and genetic material derived from the bacteria themselves, as well as from the host, and function as a reservoir for microorganisms during host colonization and also by attaching to abiotic surfaces, thereby contributing to the pathogenesis of countless species of bacteria (Donlan, 2001; Marks et al., 2014; Rabin et al., 2015). In fact, it is estimated that 65-80% of all bacterial infections are biofilm-related (Fux et al., 2005; Majik and Parvatkar, 2014), either directly or by acting as a reservoir from which virulent organisms can seed off (Bjarnsholt et al., 2013). Within the human host, most biofilms are formed by more than one bacterial species; the bacteria most commonly found in mixed communities with *K. pneumoniae* are *Pseudomonas aeruginosa* and *Pseudomonas protegens* (Periasamy et al., 2015; Joshi et al., 2021).

Studies of bacterial biofilm formation on non-living surfaces, of relevance for persistent infections through medical devices and implants, have been gaining more attention in the last 15 years (Macleod and Stickler, 2007; Monds and O'Toole, 2009; Kania et al., 2010; Krzysciak et al., 2014; Martin et al., 2019) and show that biofilm formation, due to its organization and changes in bacterial phenotypes, is a major cause of antibiotic resistance (Chao et al., 2014; Nirwati et al., 2019)

Bernier et al. demonstrated the importance of biofilm for the development of antimicrobial resistance in *E. coli*; in that study, amino acid starvation was highly associated with increased antibiotic resistance in the biofilm phenotype, but not for planktonic bacteria, reinforcing the importance of biofilm for bacterial survival in hostile environments (Bernier et al., 2013).

### Biofilm Formation and Dispersal

The process of biofilm formation includes several well-orchestrated events, starting with attachment to the colonizing surface, and continuing with production of microcolonies, biofilm maturation and further organization and finally, detachment of planktonic cells that can spread and colonize or infect sites nearby or at distant sites (Chao et al., 2014). In the attachment process, the microbial cells adhere to the surface through structures such as surface adhesins, as well as fimbriae and flagella (Marks et al., 2014; Piperaki et al., 2017). After adhesion, the bacteria multiply, generating microcolonies surrounded by self-produced extracellular polymeric substance (EPS), including polysaccharides, proteins, nucleic acids and lipids, which continues to be secreted throughout the maturation stage, providing the three-dimensional characteristic of the biofilm (Hall-Stoodley et al., 2004; Flemming and Wingender, 2010). In most cases, colonization of host surfaces likely results in incorporation also of host factors and molecules from other microbes in the resident microflora, including proteins and extracellular DNA that are used as a scaffold and further contributes to the biofilm three-dimensional structure (Okshevsky et al., 2015; Karygianni et al., 2020; Mirzaei and Ranjbar, 2022). The voids are mainly filled with water, functioning as a drainage system of waste and also acting as channels for nutrient acquisition and distribution between the micro communities (Flemming and Wingender, 2010; Jamal et al., 2018).

The dispersal step involves phenotypic changes in a fraction of the bacterial community, which detach from the structure and become planktonic. The microbial communities positively regulate the expression of proteins related to motility structures such as flagella and produce different sacrolytic enzymes that contribute to surface detachment, in response to signals from the environment, such as mechanical stress, nutrient availability, temperature variations, and the presence of extracellular ATP and other damage associated molecular patterns (DAMPs) (Baselga et al., 1994; Chao et al., 2014). Biofilm bacteria are less affected and killed than planktonic bacteria by antimicrobials and host defense systems (Guilhen et al., 2016); therefore, biofilms are regarded as a reservoir of bacteria during host infection.

### Multispecies Biofilms

It has become more and more evident that bacteria on medical devices or in host niches co-exist and synergize in polymicrobial biofilm communities (Elias and Banin, 2012; Flemming et al., 2016; Roder et al., 2016). These interactions often lead to an increased resistance against host and antimicrobial agents for all species involved (Fux et al., 2005; Attinger and Wolcott, 2012; Kong et al., 2016; Wang et al., 2022). This occurs either through increased tolerance or adaptive resistance resulting from inter-species synergy or by antibiotic-resistant species in a polymicrobial biofilm protecting other species in the biofilm against antibiotic treatment (Chatterjee et al., 2014; Puca et al., 2021). Species in polymicrobial biofilms often show increased virulence (Pastar et al., 2013), an increased ability to degrade and utilize organic compounds in their environment (Yoshida et al., 2009; Elias and Banin, 2012), and provide an environment for intra- and interspecies spread of adaptive traits and antimicrobial resistance genes (Roberts and Kreth, 2014).

The complex oral microflora may be considered one of the best studied polymicrobial biofilm environments, with well-established model systems based on physiological media, advanced imaging, and species identification by 16S rRNA gene sequencing (Bowen et al., 2018; Luo et al., 2022). Here, species composition and extracellular matrix components influence and specify the function of the biofilm by affecting cell-cell interactions and the microenvironment to modulate virulence of specific organisms (Flemming et al., 2016; Koo and Yamada, 2016). Greater diversity in the microbial community is generally associated with health (Bowen et al., 2018). Inter-kingdom interactions between bacterial and fungal species, including *Candida albicans*, are often observed in this niche and have also been well studied (Lohse et al., 2018; Ponde et al., 2021). Fungal-bacterial interactions are also important during skin and lung infections and in the gastrointestinal system, where *Candida* often helps to protect anaerobic species (Ponde et al., 2021).

Dual and polymicrobial interactions are also observed during wound infections (Goodwine et al., 2019; Warrier et al., 2021) and have been associated with urinary tract infections in patients originating from biofilms detected on urologic devices (Chatterjee et al., 2014). In the latter study, data from all culture positive catheters showed that the majority were positive for two or more bacterial species. *Pseudomonas aeruginosa*, *Escherichia coli* and *K. pneumoniae* were the most commonly isolated agents and found to co-exist (Chatterjee et al., 2014).

### *Klebsiella pneumoniae* Mixed Biofilms

Besides studies investigating single-species biofilms with *K. pneumoniae*, described in detail below, several investigations have been conducted to understand the interaction with other relevant organisms. *K. pneumoniae* and *P. aeruginosa*, two organisms that often co-exist in the environment and potentially also in the gastrointestinal tract, synergize to form biofilms with distinct structures from their respective mono-biofilms (Lee et al., 2014). Mixed biofilms of these two organisms also display increased resistance to antibiotics, such as tobramycin, as well as resistance to detergent treatment (Lee et al., 2014). These interactions were not affected by the *P. aeruginosa* quorum sensing systems Las and Rhl (Subramoni et al., 2021) but optimal biofilm formation required the Pseudomonas type IV pilus, involved in motility as well as the ability of *K. pneumoniae* to produce extracellular matrix as a non-mucoid variant of *K. pneumoniae* grew poorly in the presence of *P. aeruginosa* (Booth and Rice, 2020). In another study, carbapenemase-resistant *K. pneumoniae* were examined in the context of environmental biofilms collected from sinks in patient rooms at hospital wards (Santiago et al., 2020). Using phage therapy, the authors were able to show that a phage cocktail was able to kill *K. pneumoniae* selectively without affecting the environmental bacteria.

## Factors Contributing to *K. pneumoniae* Biofilm Formation

**Figure 1:**
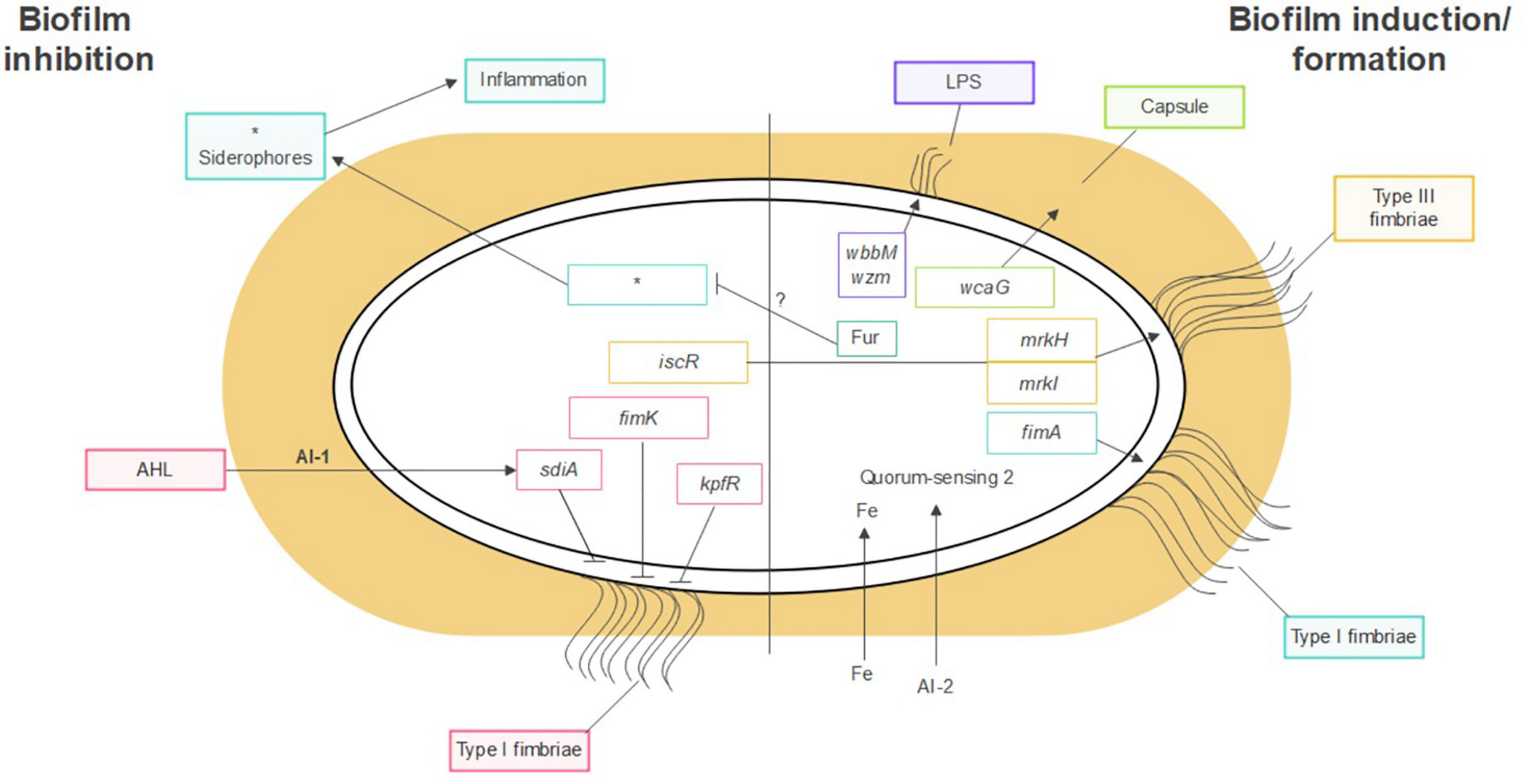
Factors contributing to *K. pneumoniae* biofilm formation. Polysaccharide capsule, LPS, fimbriae, pili, iron metabolism and, quorum sensing. *Molecules related to siderophores.

*K. pneumoniae* forms biofilms on abiotic surfaces such as medical devices and catheters, as well as on host tissues like the respiratory, urinary, and gastrointestinal tract mucosa. Several factors contribute to biofilm formation in *K. pneumoniae*. These include, among others, the polysaccharide capsule, fimbriae and pili, iron metabolism, and the presence of different bacterial species, as shown on **Figure 1**.

### Capsule

The polysaccharide capsule is an important protection mechanism for the bacterium that inhibits complement deposition and prevents bacterial opsonization and phagocytosis (Piperaki et al., 2017). It is also a highly variable structure. To date, 134 distinct capsule synthesis loci (K-loci) have been identified *K. pneumoniae* isolates through genome sequencing and comparative genomics (Wyres et al., 2016).

The polysaccharide capsule has been shown to influence different stages of biofilm formation in *K. pneumoniae*, including initial surface adhesion and maturation (Balestrino et al., 2008). Mutant strains with defects in capsule production displayed impaired biofilm formation (Zheng et al., 2018). The contribution of capsular polysaccharides to biofilm formation has been confirmed in further studies. A study evaluating biofilm formation in *K. pneumoniae* bacteremia strains found a positive association with the expression level of the virulence gene *wcaG*, involved in capsule biosynthesis (Zheng et al., 2018). Moreover, *wcaG* silencing led to a reduction in biofilm formation in these bacteria. Interestingly, the hypermucoviscosity phenotype – a common trait in bacteremic *K. pneumoniae* – was not associated with increased biofilm formation in these strains (Zheng et al., 2018).

Two other genes associated with polysaccharide production, *treC* (an enzyme that splits trehalose-6-phosphate into glucose and glucose-6-phosphate) and *sugE* (which is predicted to encode an inner-membrane protein with a very short tail facing the cytoplasm), influence biofilm formation by *K. pneumoniae* isolated by pyogenic liver abscess (Wu et al., 2011). The same study described a role for *treC* during colonization of the gastrointestinal tract, suggesting that biofilm formation is important for successful colonization and further infection (Wu et al., 2011).

In addition to their direct contribution to biofilm formation, capsular polysaccharides from *K. pneumoniae* display anti-biofilm properties against other bacteria, providing a competitive advantage in mixed bacteria environments (Goncalves Mdos et al., 2014). Furthermore, the anti-biofilm activity of the capsular polysaccharides towards other bacteria was ubiquitous and not dependent on the *K. pneumoniae* serotype.

Studies on the capsule interference in fimbriae-mediated biofilm formation show that the adhesive properties of fimbriae are influenced by capsule expression, as the presence of the capsule masks the fimbriae, reduces adhesion and consequently impairs biofilm formation (Schembri, Dalsgaard, and Klemm 2004; Schembri et al., 2005).

In a study that investigate the contribution of the capsule in *K. pneumoniae* fitness under different growth conditions, Buffet et al, showed that in a poor nutrient environment, the encapsulated strains had a fitness advantage while in a nutrient rich environment the presence of the capsule represented a disadvantage. To further highlight the correlation between capsule and biofilm formation, this study also claimed that non-encapsulated strains showed more adherence in all environments, while the capsule production of the encapsulated strain masked the fimbriae and reduced biofilm formation. However, they suggest that the ability to form biofilm is not affected by the presence of the capsule, but that amount of capsule production impairs biofilm formation (Buffet, Rocha, and Rendueles 2021).

### LPS

Lipopolysaccharide (LPS) is an important component of the external membrane in Gram-negative bacteria, including *K. pneumoniae.* The LPS also contributes to *K. pneumoniae* biofilm formation. Balestrino et al. demonstrated that LPS promotes the initial attachment of *K. pneumoniae* to abiotic surfaces and, therefore, is a critical factor in the early phases of biofilm formation (Balestrino et al., 2008). The authors showed that *K. pneumoniae* mutant strains lacking genes associated with LPS biosynthesis (*wbbM* gene) or transport (*wzm* gene) present a delay in biofilm formation. Their hypothesis is that LPS charge is necessary for the correct folding of Type 1 pilli which may explain their observation. More recently, Vuotto et al. emphasized the involvement of the *wbbM* and *wzm* genes in *K. pneumoniae* biofilm production by showing an upregulation of both genes in *K. pneumoniae* grown as biofilms in comparison with the expression in planktonic *K. pneumoniae* cells during the exponential phase (Vuotto et al., 2017).

### Fimbriae

*K. pneumoniae* expresses two main classes of fimbriae, named type I and type III. Fimbriae function as adhesins, promoting binding to biological surfaces (with subsequent tissue invasion) but also to abiotic surfaces, including medical devices, where the bacteria form biofilm (Piperaki et al., 2017). The genome of *K. pneumoniae* harbors at least 10 clusters of genes encoding chaperones, ushers, and adhesin proteins for the assembly of fimbriae, including *fim*, *mrk*, *ecp* and *kpa* to *kpg* gene clusters (Wu et al., 2010; Alcantar-Curiel et al., 2013; Khater et al., 2015). Among these, the most experimentally well-characterized are *fim*, *mrk*, *ecp* and *kpf*, which encode type I and type III fimbriae (Paczosa and Mecsas, 2016), common pilus (Alcantar-Curiel et al., 2013), and the type I-like fimbriae (Gomes et al., 2020).

The two main classes of fimbriae expressed by *K. pneumoniae* are type I and type III fimbriae. Type I fimbriae bind to receptors containing mannose, found in various tissues in the human host (Klemm and Schembri, 2000). Type III fimbriae have been shown to bind to different cell types *in vitro*, including kidney, lung and bladder epithelial cells. Although MrkD – the adhesin in type III fimbriae – has been shown to interact with collagen structures (Jagnow and Clegg, 2003), the specific cellular receptor for this molecule has not been identified.

In terms of biofilm formation, the type III fimbriae appear to be consistently associated with increased biofilm formation, whereas the role of type I fimbriae appears to be more complex and varies depending on experimental conditions and host niche. A study evaluating 33 strains of *K. pneumoniae* found a positive correlation between type III fimbriae expression and biofilm formation on abiotic surfaces, while a strain expressing only type I fimbriae did not form biofilms (Di Martino et al., 2003). This data was corroborated by Schroll et al. who found that type III, but not type I fimbriae, increase biofilm formation in a flow-cell system (Schroll et al., 2010). They also found that a mutant strain lacking type I fimbriae was not affected in its ability to form biofilms. Furthermore, in biofilms formed with bacteria lacking type III fimbriae, the fim operator was OFF, suggesting repression of type I fimbriae expression under biofilm conditions (Schroll et al., 2010). Also, the role of type III fimbriae in biofilm formation is supported by the observation that mutant *K. pneumoniae* strains lacking this gene cluster produced significantly less biofilm in the presence of lung surfactant, with cholesterol and phosphatidylcholine identified as surfactant components promoting type III fimbriae expression (Willsey et al., 2018). This study investigated factors of importance for survival in the lung and did not identify a role for Type I fimbriae. These studies combined would indicate a limited role of type I fimbriae in biofilm formation.

However, in contrast, a later study found that both type I and type III fimbriae promote biofilm formation in a model of catheter associated bladder infection using human urine as a culture medium (Stahlhut et al., 2012). In this study, only the double-negative mutant was significantly attenuated in its ability to form biofilms. Also, high expression of both fimbrial types was detected in mature 70 h biofilms, indicating that these structures are important also during later stages of biofilm development or maintenance (Stahlhut et al., 2012). Interestingly, immunostaining of biofilm bacteria using specific antibodies revealed that type I and type III fimbriae are not expressed simultaneously, suggesting overlapping roles for bacterial virulence. Finally, transformation of non-fimbriated *E. coli* with plasmids carrying either type I or type III fimbriae promoted an increase in biofilm formation on catheters, reinforcing the individual contribution of these structures to biofilm formation.

As the production of type I and III fimbriae has been shown to impact biofilm formation, how these fimbriae are regulated has important implications for the early stages of bacterial adhesion that lead to biofilm development. Regulation of fimbriae expression is an intricate process influenced by factors such as iron availability, oxidative stress and DNA binding regulators (Wu et al., 2012; Ares et al., 2016). Regarding the regulation of type III fimbrial genes and biofilm formation, Johnson et al. showed that the MrkH and MrkI regulators positively control the expression of type III fimbriae in *K. pneumoniae*, while mutant strains lacking both these regulators have a significantly decreased ability to form biofilms (Johnson et al., 2011). The *mrkH* and *mrkI* genes encode for proteins with a c-di-GMP-binding PilZ domain and a LuxR-type transcriptional regulator, and their regulatory effect is mediated by c-di-GMP. In the presence of this effector molecule, MrkH activates transcription of the MrkA promoter, leading to type III fimbriae expression and increased biofilm production (Wilksch et al., 2011). Also, Lin et al. showed that the global regulator IscR represses the expression of type III fimbriae in *K. pneumoniae* and strains depleted of IscR show an increased ability to form biofilms (Lin et al., 2017). According to those authors, IscR represses type III fimbriae by directly repressing the synthesis of MrkH and MrkI regulators (Johnson et al., 2011; Lin et al., 2017).

Type I fimbriae expression is controlled at the transcriptional level by phase variation and by the action of transcriptional regulators. Phase variation is mediated by an invertible DNA segment, named the *fimS* element, which contains the promoter of the *fim* operon and whose orientation determines the fimbriated or the nonfimbriated phenotypes (Abraham et al., 1985). The ON-orientation, corresponding to activation of fimbriae expression, was found in *K. pneumoniae* infecting the bladder, while the OFF-orientation was prevalent during gut and lung colonization with the same strain (Struve et al., 2008). This result suggests that type I fimbriae expression is influenced by the colonizing site and environment during infection. In addition to the phase variation, two type I fimbriae repressors, FimK and KpfR, represent inhibitory factors for biofilm formation by *K. pneumoniae*, since loss of *fimK* or *kpfR* genes renders mutant strains with a hyperfimbriated phenotype and an enhanced ability to form biofilms (Rosen et al., 2008; Gomes et al., 2020). FimK harbors an EAL domain in its carboxy-terminal region and it is involved in cleavage of c-di-GMP (Rosen et al., 2008). The presence of FimK was also associated with impaired adherence to the bladder of *K. pneumoniae* in comparison with UPEC in the early stages of infection, due to the inhibition of fimbriae production. However, the mechanism of fimbriae inhibition by FimK, as well as the factors regulating its action, are unknown. The *kpfR* gene encodes a transcriptional regulator of the *kpf* gene cluster, which encodes for a type I-like fimbriae. Our group has shown that KpfR expression is regulated by Fur (ferric uptake regulator) through an iron-dependent mechanism (Gomes et al., 2020).

Besides FimK and KpfR, our group has recently described a quorum-sensing regulator, SdiA, which controls the expression of virulence factors, including type I fimbriae, and affects biofilm formation (Pacheco et al., 2021). Mutant strains lacking SdiA showed increased biofilm formation *in vitro*, which correlates with an up-regulation of type 1 fimbriae, suggesting that SdiA acts as a repressor for fimbriae production and biofilm formation.

In addition to type I and III fimbriae, the two other fimbrial gene clusters experimentally characterized in terms of biofilm formation and cell adherence were the *ecp* and *kpf* gene clusters. The *ecp* operon is composed of the *ecpR-A-B-C-D-E* genes and encodes ECP, a pilus homologous to the *Escherichia coli* common pilus that is required for biofilm formation (Alcantar-Curiel et al., 2013), The *kpf* gene cluster comprises the *kpfR-A-B-C-D* genes and encodes type I-like fimbriae (Wu et al., 2010; Gomes et al., 2020). *K. pneumoniae* cells depleted of *kpfR* gene, which encodes a transcriptional repressor of fimbrial expression, present up-regulation of both type I and type I-like fimbriae, resulting in enhanced biofilm formation and greater adhesion to epithelial host cells (Gomes et al., 2020)

In an attempt to better understand the systems of chaperones, ushers, and adhesins encoded by *K. pneumoniae* for fimbriae assembly, Khater et. al, managed to construct and characterize mutant *K. pneumoniae* cells for the usher-encoding genes from each of the *kpa* to *kpg* fimbrial loci (Khater et al., 2015). According to the authors, only the deletion of the usher-encoding genes from *kpa* and *kpg* loci results in a significant reduction in biofilm formation compared to the wild-type strain. Moreover, only the strain depleted of the usher-encoding gene from the *kpg* adheres less to human cells than the wild-type strain (Khater et al., 2015). The broad number of chaperone, usher, and adhesin encoding genes on the *K. pneumoniae* genome highlights the impressive adhesion capacity of this bacterium and emphasizes the need to deepen the understanding of the colonization ability of *K. pneumoniae*.

In summary, *K. pneumoniae* fimbriae play important roles during biofilm formation, which vary according to the infection site (or abiotic surface) and are not limited to initial attachment of bacteria. Regulation of fimbriae expression is a complex process controlled by several regulators, which respond to diverse environmental signals in different host niches, while the absence of co-expression of type I and type III fimbriae suggests a co-regulation system with mutual inhibitory effects.

## Regulation of Biofilm Formation

### Iron Metabolism

In pathogenic bacteria, one signal that triggers infection and colonization is the iron deprivation that the pathogen faces when in contact with the host. In fact, iron plays a crucial role in the regulation of numerous virulence factors, as well as biofilm formation (Gomes et al., 2020). Pathogens obtain iron through the activation of iron uptake systems. One of the most effective strategies to acquire iron from mammalian hosts is through the production of iron chelator molecules, named siderophores, which have a high affinity for iron. *K. pneumoniae* has four siderophore-mediated iron uptake systems, mediated by the enterobactin, yersiniabactin, salmochelin, and aerobactin siderophores (Fang et al., 2000; Dao et al., 2014; Paczosa and Mecsas, 2016). Enterobactin has the highest affinity for iron and it is widely spread among *K. pneumoniae* strains. On the other hand, aerobactin and salmochelin are typically found in hypervirulent *K. pneumoniae* strains and represent critical virulence factors in these strains ( (Russo et al., 2014; Paczosa and Mecsas, 2016).

Guilhen at al. found that the expression of all siderophore-encoding genes are greatly down-regulated in *K. pneumoniae* biofilm-dispersed and sessile cells compared to planktonic cells (Guilhen et al., 2016). In a separate study, Guilhen at al. reported that biofilm-dispersed and sessile cells elicit a lower innate immune response than planktonic cells. Taking both results together, the authors suggest a possible immune evasion strategy of biofilm-dispersed cells by repressing siderophore genes. Since siderophores are known to induce strong pro-inflammatory responses, by producing fewer siderophores the biofilm-dispersed cells would hide from the immune system by avoiding overstimulation of the immune response (Guilhen et al., 2019).

The influence of iron levels on biofilm development is also evidenced by studies conducted using iron chelators and antagonists. For instance, Hancock et al. demonstrated that biofilm formation by uropathogenic *E. coli* strains is impaired in the presence of divalent metal ions such as Zn(II) and Co(II) (Hancock et al., 2010). Similarly, Chhibber et al. (2013) investigated the ability of *K. pneumoniae* to form biofilm in the presence of divalent Co[II] ions (an iron antagonizing ion), in combination with a bacteriophage encoding depolymerase enzyme that degrades exopolysaccharides on the biofilm structure. The authors observed a significant reduction in biofilm formation by *K. pneumoniae* when both elements were present (Chhibber et al., 2013). According to Chhibber and collegues, the combination of Co[II] and depolymerase producing phage caused an inhibition of biofilm formation as well as a disruption of mature biofilms, suggesting a possible adjuvant effect in treatment of persistent, biofilm forming *K. pneumoniae* infections. Finally, Chen et al. (2020) demonstrated that liver abscess-causing *K. pneumoniae* strains grown in an iron-supplemented medium had a strong biofilm formation, whereas the addition of the iron chelator 2,2’-Dipyridyl resulted in decreased growth and inhibited biofilm formation.

In *K. pneumoniae*, as in most bacteria, the iron homeostasis is controlled by the transcriptional regulator Fur (Gomes et al., 2020). Fur exerts transcriptional activation or repression of target genes by either forming a complex with its cofactor, iron, or remaining in its apo-form (no cofactor bound) (Escolar et al., 1999; Gomes et al., 2020). Considered a global transcriptional regulator, Fur modulates the expression not only of genes related to iron metabolism, but also numerous genes associated with virulence factors, including some involved on biofilm development (Troxell and Hassan, 2013). For instance, Fur plays a role, in the presence of iron, in biofilm formation by regulating the expression of type I (Gomes et al., 2020; Pacheco et al., 2021) and type III (Wu et al., 2012) fimbrial genes, and also the expression of transcriptional regulators of fimbriae (Wu et al., 2012; Gomes et al., 2020).

### Quorum Sensing

All the steps of biofilm formation are regulated by signaling molecules, which are part of the quorum sensing system (Guilhen et al., 2019; Saxena et al., 2019). Due to varying environmental stimuli, the bacterium inside the biofilm needs to be able to quickly adapt and change gene expression, therefore the transcriptional regulation is also controlled by quorum sensing (Wang et al., 2020).

Quorum sensing (QS) is a sophisticated mechanism that allows communication between bacteria of the same species or between different species within the same community. Communication is based on the production, secretion, and detection of small molecules called AutoInducers (AI) (Balestrino et al., 2005). When the concentration of these molecules in the extracellular matrix reaches a threshold, the signal is detected by the bacteria and induces a change in the expression of certain genes, modifying the bacterial phenotype, expression of virulence factors, acid tolerance and biofilm formation (Balestrino et al., 2005; Saxena et al., 2019). Besides its role in coordinating biofilm formation, quorum sensing is also important for the maintenance of mature biofilms (Abraham, 2016).

Two main types of QS communication systems have been described on Gram-negative bacteria (Miller and Bassler, 2001). The type 1 QS is primarily used for intra-species communication and uses acyl-homoserine lactones (AHL) as autoinducers of type 1 (AI-1). AHL binds to its cognate receptor, the transcriptional regulator LuxR, to regulate the transcription of target genes. *K. pneumoniae* does not produce AI-1 molecules (Balestrino et al., 2005), but encodes SdiA, a LuxR-type receptor that, interestingly, responds to AHL molecules synthesized by other bacterial species (Pacheco et al., 2021). The type 2 QS allows intra- and inter-species communication and involves cyclic furanone compounds as autoinducers of type 2 (AI-2). LuxS synthase is the key enzyme in AI-2 molecules production, and a homolog of the *luxS* gene has been identified in the *K. pneumonia*e genome (Balestrino et al., 2005).

Previous studies have demonstrated the regulatory effect of both type 1 and type 2 QS in biofilm formation by *K. pneumoniae*. Firstly, Balestrino et al. revealed that a *luxS*-deficient mutant strain of *K. pneumoniae* shows a reduced capacity to develop microcolonies during biofilm development, although the strain is still able to form a mature biofilm (Balestrino et al., 2005). These data suggest that AI-2 molecules play a role in the early steps of biofilm formation.QS also regulates *K. pneumoniae* biofilm formation by modulating the expression of genes that play an import ant role in biofilm development, such as genes encoding bacterial surface structures (including fimbriae) and exopolysaccharides (such as LPS). According to De Araujo *et al.*, *K. pneumoniae* strains deficient in both AI-2 synthesis and transport show increased expression of LPS related genes, leading the mutant strains to form a biofilm with greater biomass, although with altered architecture. The authors hypothesized a role of AI-2 molecules in regulating biofilm formation and LPS biosynthesis in sessile *K. pneumoniae* cells (De Araujo et al., 2010).

Quorum sensing and intra- and interspecies communication is crucial for coordinated behaviors of bacteria in biofilm communities at both those consisting of single species and polymicrobial biofilms (Thompson J. A. et al., 2015; Mukherjee and Bassler, 2019; Warrier et al., 2021). Quorum sensing systems are responsible for multiple coordinated events, including virulence gene expression (Bronesky et al., 2016), metabolite production and use (Barnard et al., 2007), as well as competence and DNA uptake and integration and spread of fitness traits and resistance genes (Marks et al., 2014).

## Models of Biofilm Evaluation

The simplest *in vitro* model for biofilm evaluation is the microplate assay. In this model, the bacterium is diluted and added to a polyestyrene plate, which is incubated for various time periods. *K. pneumoniae* usually forms biofilms at the liquid-solid interface, being more attached to the well bottom, and the biofilm mass is evaluated after coloration using crystal violet. The result is shown as the optical density measured after solubilization of the crystal violet (CV)-stained biofilm structure using either ethanol or 30% acetic acid. In this method, more biomass (more biofilm) is represented by higher absorbance, since more CV will be retained by the cells. Several studies have used this cheap and straight-forward technique (Singh et al., 2019; Chen et al., 2020; Townsend et al., 2020; Pacheco et al., 2021).

Due to its easy execution, the CV model has been vastly used to analyze the bacterial biomass; however, this technique has important limitations, since it does not provide data on the quality of biofilm and its structure.

In a variation of the microplate model, Lalitha et al. used a coverslip inserted into the culture plate, which was incubated with bacteria for 72 hours, removed from the plate, and analyzed by microscopy, thus providing structural information (Lalitha et al., 2017). Another model used by the same group evaluated biofilm formation on a silicone catheter, which was cut into 2 × 2 cm catheter discs, added to the culture plate and incubated with bacteria for 72 hours. The biofilm was formed on the discs, which were either analyzed by microscopy or vortexed and the mechanically dispersed biofilm bacteria were plated for CFU counting (Lalitha et al., 2017). This model is a good way to analyze the biofilm structure using microscopy, since the biofilm is formed just like in the microplate, but the coverslip can be removed for microscopy analyses, however it makes it difficult to assessing for gene expression and other molecular biology tests.

Since biofilm formation in catheters are a main cause of urinary tract infection, a model to analyze the biofilm on this structure was developed and adapted by several groups. A study by Desai at al. compared biofilm formation using 3 kinds of catheters: latex, silicone and silicone coated latex, and 3 types of culture medium: Luria-Bertani (LB), artificial and natural urine. They found that latex catheters, the cheapest and most used model in patients, as well as natural urine were the substrates that stimulated biofilm formation most effectively (Desai et al., 2019).

More recently, Townsend et al. have published a study where they used a Foley silicone coated latex urinary catheter to assess the biofilm inhibition by bacteriophages and antimicrobials (Townsend et al., 2020); their model consists of incubating the bacterial suspension with 1,5 cm cut catheter pieces and allow the biofilm to form (around 16 hours); after that time, the biofilm is exposed to the phage cocktail and/or the antibiotic. They found that phage cocktails have the potential to prevent *Klebsiella* biofilms in catheters, if used early or as a preventative strategy (Townsend et al., 2020).

A more physiological model to study biofilm formation on a urethral catheter, using a bladder-like system mimicking the steps of a natural urinary infection, was developed by Stickler and colleagues. The system is composed of two glass compartments (an inner and outer compartment) maintained at 37°C by a water jacket and a balloon filled with human urine, which was inserted inside the inner compartment to mimic a bladder. A catheter was then inserted together with the bacterium in the balloon and biofilm was formed on the catheter. The study was used to establish a novel biofilm model for different bacterial species. (Stickler et al., 1999). This model is interesting, as the idea is to mimic a real bladder biofilm and simulate a real *in vivo* infection, however, it requires a very specific apparatus that should be constructed by the user and can be tricky to be reproduce in different locations, depending on the material available. Also, the use of human urine as culture medium is difficult to reproduce since the urine composition vary from person to person.

An interesting methodological approach was described by Cubero et al. to evaluate the biofilm formation by a K1 hypermucoviscous phenotype strain, through an adapted protocol previously used to assess the phenotype of a different bacterium in an air-liquid interface (Nait Chabane et al., 2014). The model consists of growing a single bacterial colony in 12 mm diameter polystyrene tubes in Brain Heart Infusion (BHI) broth, at 37°C for 72 hours. The biofilm is identified by a thick pellicle covering the liquid surface; the pellicle is strong enough to sustain the whole medium weight when the tube is inverted (Cubero et al., 2019). It is a simple model and easy to apply, but it is restricted to the K1 serotype, since it demands that the strain presents the hypermucoviscous phenotype.

An *in vitro* model that has been used by several authors is the continuous flow model; in this model, the bacterium is incubated inside a bioreactor, where the medium is continuously pumped at a fixed flow rate and the bacterium is allowed to form biofilm for longer time periods. In a recent study, Touzel et al. established a model of polymicrobial biofilm that mimics the wound conditions of diabetic ulcers to test the ability of chlorhexidine, a largely used antiseptic, to penetrate biofilms (Touzel et al., 2016). In this model, the authors have grown four bacteria: *Staphylococcus aureus*, *Enterococcus faecalis*, *Pseudomonas aeruginosa* and *Klebsiella pneumoniae* as a mixed biofilm in a continuous flow bioreactor, at 37°C for 30 h. In addition, the medium was supplemented with 5% horse blood to simulate the wound environment. The study showed that chlorhexidine does not effectively penetrate the structure of the biofilm (Touzel et al., 2016).

Another continuous flow model was described by Stewart et al. The authors used an annular reactor to evaluate the biofilm formation by two species, *Pseudomonas aeruginosa* and *Klebsiella pneumoniae*. In this model, the bacteria form biofilm in stainless steel slides inside the bioreactor. At specific time points, the slides were removed for cell count and the biofilm structure was evaluated using fluorescent antibodies and confocal microscopy. As a result, *K. pneumoniae* outcompeted *P. aeruginosa* due to its high growth rate (Stewart et al., 1997). An advantage of the continuous flow models is that bacteria can grow on the bioreactor for longer time periods, allowing for better biofilm development.

Other physiological models to evaluate biofilm formation use cells as a substrate for biofilm growth. In these models, different cells can be grown util confluence and fixed to serve as a bed substrate for the bacteria to grow and form biofilms. Ostria-Hernandez and colleagues have used human lung Calu-3 cells and compared the biofilm formation between abiotic (just the plastic plate) substrate and biotic substrate. They found that biofilm formation on an abiotic substratum was much higher than the biofilm formed on the Calu-3 cells. They hypothesized that as they evaluated bacterial strains isolated from hospital-acquired infections, these strains may likely be more adapted to colonize abiotic surfaces than community-acquired strains (Ostria-Hernandez et al., 2018). Jagnow and Clegg described two models to evaluate biofilm formation. In one model they used coverslips coated by fixed human bronchial epithelial (HBE) cells as substrate and in the other model, they coated the coverslip with collagen solution to show the importance of the adhesins MrkA and MrkD for biofilm formation, particularly MrkD which possess collagen-binding domains (Jagnow and Clegg, 2003). More recently, Guilhen et al, studied biofilm formation on top of two cell lines, the lung carcinoma cell line A549 and the pharyngeal epithelial cells FaDu. These cell substrates were used to compare the colonization between planktonic and biofilm dispersed bacteria and the authors showed that the biofilm dispersed bacteria are more efficient in colonizing both biotic and abiotic (plastic plates) surfaces than the planktonic bacterium on both substrates (Guilhen et al., 2019). These models are considered more appropriate and physiological in the study of biofilm formation during colonization of mucosal surfaces because they mimic the host environment and therefore stimulate the same molecular interactions found during natural colonization.

In terms of *in vivo* biofilm models, Murphy *et al.* described a urinary tract infection model using mice, which were anesthetized and inoculated through the transurethral route with bacteria, followed by insertion of a silicone tube into the bladder. This model was used to study the role of type I and type III fimbriae during biofilm formation (Murphy et al., 2013). Another mouse model was described by Guilhen and colleagues, where the authors compared the immune response elicited by mouse infection with planktonic and biofilm dispersed bacteria. The animals were infected *via* the intranasal route and the colonization and bacterial burden was assessed in the lungs and cytokine production was analyzed from the spleen 6, 24 or 48 h after the infection. They found that the biofilm-dispersed bacteria induced less pro-inflammatory cytokine production in comparison to the planktonic bacteria (Guilhen et al., 2019).

Another interesting model was described by Thompson et al., who have developed a dorsal wound model to investigate biofilm formation in mice (Thompson M. G et al., 2015). They used the model to test the effect of a topical formulation containing gallium citrate (GaCi) for the treatment of wounds infected with *K. pneumoniae.* The model consists of using a 6.0-mm disposable skin biopsy punch to cave a wound on the animal back, then 25 microliters containing the bacterium are inoculated in the wound, which is monitored until day 3 post inoculation for signs of infection and healing. The authors found that mice treated only with placebo developed a well-structured biofilm and those treated with 0.1% gallium citrate (GaCi) formed less biofilm, showing its antibiofilm properties, which may be a promising alternative to prevent and/or treat *K. pneumoniae* biofilms (Thompson M. G et al., 2015). This is a great model to study biofilm formation during skin infections, which is also particularly interesting for skin pathogens such as *Staphylococcus aureus*.

A rabbit model was described by Gurjala et al. to evaluate biofilm formation during skin infection. A 6 mm wound was carved on the anesthetized rabbit’s ears, which was then infected with *K. pneumoniae*. Biofilm development was analyzed after different time points, using microscopy of the tissue to analyze the biofilm formation in the wounds (Gurjala et al., 2011).

Finally, an interesting model was described by Benthall *et al*. They used an insect larvae model to evaluate the bacterial resistance to different antibiotic treatments. Using the greater wax moth (*Galleria mellonella*) larvae, they induced infection using a toothbrush bristle cultured together with the bacterial strain for 24 hours to allow biofilm to form on the bristle. The bristle was then inserted into the proleg of *G. mellonella* larvae and antibiotics were administered 30 min after the infection in a different proleg (Benthall et al., 2015). The authors applied this protocol to compare the antibiotic resistance of the *K. pneumoniae* strain *in vitro* (using a MIC assay) and *in vivo*, and they showed that results from both protocols corresponded well, with the exception that in the *Galleria* model the carbepenemase test appears to perform better than it does *in vitro.* Additionally, they infected the *Galleria* larvae with planktonic or biofilm bacteria in the presence of two antibiotics (amikacin and ciprofloxacin) and compared the outcomes. The results showed that the larvae infected with planktonic bacteria had a higher survival rate than the biofilm infected larvae for both antibiotics (Benthall et al., 2015).

The *Galleria*, however, is a controversial model. Bruchmann and colleagues, have recently published a study where they explore this model to identify genes related to MDR strains, and the model showed itself very useful and the authors were able to describe several genes related to virulence factors that are involved in biofilm formation in MDR strains (Bruchmann et al., 2021). On the other hand, Russo and Macdonald have applied two models to evaluate the difference between hypervirulent and classic (less virulent) *K. pneumoniae* strains, and found that the mouse infection model was much more accurate than the *Galleria* model to differentiate the two groups which represent a disadvantage for this model (Russo and MacDonald, 2020).

Although there are several different *in vitro* models to study biofilm formation in *K. pneumoniae*, they all have inherent limitations that may impact the correlation with natural infections in the host. On the other hand, few animal models have been developed, which represent a gap in the ability to study the influence of biofilms during pathogenesis. *In vitro* models are simple to use and can be used to screen mutants and can easily be controlled for different types of host environmental factors to explore and identify novel mechanisms. By making them as physiological as possible, to mimic the environment during infection, a better prediction of how the bacteria may behave *in vivo* can be done. *In vivo* models provide better tools to understand virulence strategies but are expensive, complex, and difficult to vary to understand mechanisms. Both types of models are needed, serves important purposes, and have their pros and cons. They are both necessary for future biofilm research and the choice of models that the researcher uses should be based on what models provide the best answers for their specific research questions.

## *K. pneumoniae* Biofilms as Vaccine/Therapeutic Targets

Many factors involved in biofilm formation have been investigated as potential vaccine candidates against *K. pneumoniae* infection, with a focus on the anti-biofilm properties of the induced antibodies. A formulation comprising immunodominant epitopes of outer membrane proteins, called AK36, was investigated in a mouse model of systemic infection. Immunization with the protein chimera induced the production of antibodies capable of limiting biofilm formation by *K. pneumoniae in vitro*, while passive immunization with these antibodies promoted protection against intraperitoneal challenge (Babu et al., 2017). These results suggest that the ability of vaccine antibodies to prevent biofilm formation may contribute to protection also *in vivo*.

A study from Navarro et al. characterized the antibody response generated in mice injected with a mixture of capsular polysaccharides from carbapenem-resistant *K. pneumoniae* fused to anthrax protective agent (PA) protein (Diago-Navarro et al., 2018). Two monoclonal antibodies (Mabs) were able to impair biofilm formation on polystyrene plates after 16 h incubation, as well as promoting complement deposition and phagocytic killing while reducing serum resistance of the bacteria. Furthermore, mice challenged intratracheally with pre-opsonized bacteria displayed reduced bacterial dissemination to the lungs, liver and spleen (Diago-Navarro et al., 2018).

Another study investigated the anti-biofilm properties of Mabs recognizing the type III fimbriae protein MrkA, which have been selected from phage display and hybridoma platforms using functional opsonophagocytic assays (Wang et al., 2016). The anti-MrkA antibodies induced a dose-dependent inhibition in biofilm formation *in vitro* and reduced bacterial attachment to human lung epithelial cells in culture. These functional assays correlated with the increased survival of mice after intranasal challenge and lower bacterial dissemination. In the same study, subcutaneous immunization with recombinant monomeric or oligomeric MrkA protected mice against intranasal challenge, as demonstrated by reduced organ damage in comparison with the control (Wang et al., 2016).

In summary, since biofilms play an important role in *Klebsiella* pathogenesis, the ability of antibodies to inhibit biofilm formation is one possible protective mechanism against infection with this bacterium.

## Conclusion

Biofilm formation is an important feature in *Klebsiella pneumoniae* disease pathogenesis, promoting increased resistance against environmental stressors and providing a reservoir for dissemination and further gene exchange associated with antimicrobial resistance. Several virulence factors contribute to biofilm formation by *K. pneumoniae*, either directly, by promoting increased adherence and/or biofilm maturation, or indirectly, by inhibiting biofilm formation by bacterial competitors in their colonizing niche. Many of these molecules have been studied as vaccine candidates or targets for new therapeutic agents against this bacterium (Assoni et al., 2021). Considering the complexity of *K. pneumoniae* infections, which affect different host niches, multiple models have been developed to evaluate biofilm formation, combining *in vitro* and *in vivo* assays. However, there is still a lack of *in vivo* models to evaluate the contribution of biofilm development for disease pathogenesis. In that sense, the combination of different methodologies may provide a more detailed scenario accurately reflect the steps of natural infection.

## Author Contributions

MS, BV, GD, AL, LF, AH, MD, and TC have drafted the manuscript. GD produced the figure. LF, AH, MD, and TC have revised the text. GD produce the figure. All authors read and approved the final manuscript.

## Funding

This work was supported by FAPESP by the grants: 2019/23566-6, and 2021/01211-1. Conselho Nacional de desenvolvimento Científico e Tecnológico (CNPq): 400099/2022-5. Swedish Research Council (VR) grant number: 2021-06050.

## Conflict of Interest

The authors declare that the research was conducted in the absence of any commercial or financial relationships that could be construed as a potential conflict of interest.

## Publisher’s Note

All claims expressed in this article are solely those of the authors and do not necessarily represent those of their affiliated organizations, or those of the publisher, the editors and the reviewers. Any product that may be evaluated in this article, or claim that may be made by its manufacturer, is not guaranteed or endorsed by the publisher.
