# Corrigendum: *Klebsiella pneumoniae* biofilms and their role in disease pathogenesis

**DOI:** 10.3389/fcimb.2025.1564010

**Published:** 2025-02-06

**Authors:** Maria Eduarda Souza Guerra, Giulia Destro, Brenda Vieira, Alice S. Lima, Lucio Fabio Caldas Ferraz, Anders P. Hakansson, Michelle Darrieux, Thiago Rojas Converso

**Affiliations:** ^1^ Laboratório de Biologia Molecular de Microrganismos, Universidade São Francisco, Bragança Paulista, Brazil; ^2^ Division of Experimental Infection Medicine, Department of Translational Medicine, Lund University, Malmo, Sweden

**Keywords:** biofilm, *Klebsiella pneumoniae*, quorum sensing, pathogenesis, virulence factors

In the published article, there was an error in the author list, and author Giulia Destro was erroneously missing the information that Maria Eduarda Souza Guerra and Giulia Destro equally contributed to this work and shared first authorship. The corrected author list appears below.

“Maria Eduarda Souza Guerra^1†^, Giulia Destro^1†^, Brenda Vieira^1^, Alice S. Lima^1^, Lucio Fabio Caldas Ferraz^1^, Anders P. Hakansson^2^, Michelle Darrieux^1^, Thiago Rojas Converso^1*^”.

†These authors have contributed equally to this work and share first authorship

The authors apologize for this error and state that this does not change the scientific conclusions of the article in any way. The original article has been updated.

